# Anatomical site variations in healing of pediatric femoral shaft fractures: ultrasound evaluation of conservative treatment

**DOI:** 10.3389/fped.2025.1682441

**Published:** 2026-01-05

**Authors:** Xiang Li, Junxia Song, Yuan Zhang, Jun Wu, Xing Liu

**Affiliations:** 1 National Clinical Research Center for Children and Adolescents’ Health and Diseases, Ministry of Education Key Laboratory of Child Development and Disorders, Department of Orthopedic, Children’s Hospital of Chongqing Medical University, Chongqing, China; 2 Department of Emergency Medicine, Chongqing Hospital of the Chinese People's Armed Police Force, Chongqing, China; 3 Department of Ultrasound, Chongqing Health Center for Women and Children, Women and Children’s Hospital of Chongqing Medical University, Chongqing, China; 4 International Science and Technology Cooperation Base of Child Development and Critical Disorders, Chongqing, China; 5 Chongqing Municipal Health Commission Key Laboratory of Children’s Vital Organ Development and Diseases, Chongqing, China; 6 Department of Pediatrics, Chongqing Health Center for Women and Children, Women and Children’s Hospital of Chongqing Medical University, Chongqing, China

**Keywords:** ultrasonography, femoral shaft fracture, conservative treatment, pediatric fracture, bone callus

## Abstract

**Objective:**

Ultrasonography is a new alternative to conventional x-ray in fracture examination that avoids radiation damage, but it is unclear whether it can assess fracture healing in children. In this study, we propose to utilize Color Doppler ultrasound to examine in conservative treatment for pediatric femoral shaft fractures, aiming to determine whether there were differences in bone healing at different diaphyseal sites, thereby clarifying the value of ultrasound in fracture healing assessment.

**Methods:**

We performed a prospective observational study to investigate children with femoral shaft fractures treated conservatively who were admitted to our hospital from March 2017 to December 2021. All cases were divided into three groups according to the site of the fracture: upper, middle, and lower segments, and the children were followed-up and observed using Color Doppler ultrasound at the 1st, 2nd, 3rd, and 4th weeks after the injury, recorded the callus-to-femur width ratio (callus thickness/femur width, cm/cm), callus growth rate (callus thickness/days, cm/d), and vascular Resistive Index (RI) and compared the fracture recovery between the groups.

**Results:**

This study included 31 males and 12 females, for a total of 43 children with femoral shaft fractures. The results showed no statistical difference in callus-to-femur width ratio (cm/cm), callus growth rate (cm/d), and vascular Resistance Index (RI) detected at different fracture sites at the same time after injury (*p* > 0.05). However, we found that the RI were smaller in the group with faster callus growth rate (cm/d) and vice versa. In addition, when examined at different time, there were statistical differences in the callus-to-femur width ratio (cm/cm), callus growth rate (cm/d), and RI between the different time groups (*p* < 0.05), and as the time after injury increased, the callus growth rate (cm/d) gradually became slower and the RI value gradually decreased.

**Conclusion:**

These results indicate that children with femoral shaft fractures at different anatomical sites demonstrated comparable recovery rates during the early 4-week healing period under conservative treatment, and Color Doppler ultrasound can monitor the recovery process and effectively evaluate bone callus growth by detecting the blood supply around the edge of the fracture.

## 1 Introduction

The incidence of fractures in children is about 3% (1), most commonly in preschool children, and its incidence tends to be more in males than females (2). x-ray has been widely used as the gold standard for fracture diagnosis, but it also has disadvantages such as ionizing radiation, health hazards, and a tendency to increase the risk of malignant tumor development (3, 4), and for growing children, radiation hazard may pose a greater risk (3, 5, 6). In addition, relevant studies have shown that some children may not have obvious pathological signs after the occurrence of fractures, and there may be false-negative results on plain x-ray examinations (7–11). Over the past century, the scope of ultrasound applications has continuously broadened, achieving especially remarkable progress in medical practice. In recent years, a growing body of scholars has initiated research into the applications of ultrasonography in fracture assessment (1, 7, 9, 12). Ultrasonography provides relatively good imaging of soft tissue injuries (13–15) and can assist in clarifying whether there is concomitant nerve damage (16–22), and the information can assist in diagnosis of bone fractures. Nowadays, ultrasonography is gradually being used in children with fractures to assist in localization during surgical treatment (23–26). During the healing process of fracture, the distribution of blood supply at the fracture site is extremely important (27). Vascular imaging can also be performed by using Color Doppler ultrasound, which can help further clarify fracture. In a study by Santolini (28), it was noted that the division of the femur into upper, middle, and lower parts, with moderate, high, and low levels of vascularization, respectively, would affect fracture healing to varying degrees. In this prospective observational study, we analyzed 43 children who were treated with conservative traction for femoral shaft fractures at the Children's Hospital of Chongqing Medical University (hereinafter referred to as “our hospital”), and used Color Doppler ultrasound to monitor the fracture healing process to determine whether there were differences in early bone healing period at different anatomical sites.

## 2 Material and methods

### 2.1 Setting

Children with femoral shaft fractures treated in our hospital between March 2017 and December 2021 were included in this analysis. Inclusion criteria were as follows: (1) age ≤16 years; (2) femoral shaft fractures confirmed by x-ray examination; (3) treated with conservative traction. Exclusion criteria were as follows: (1) open fractures, multiple fractures, pathological fractures or comminuted fractures; (2) surgical treatment; (3) refusal of ultrasonography; (4) incomplete follow-up.

Each patient's guardian agreed to participate in the study and the study protocol was approved by our hospital's ethics committee (No.117;2019). The data in the study were obtained from our hospital records.

### 2.2 Instrument and preparation

The Philips-CX50 color Doppler ultrasound diagnostic instrument (Philips, Amsterdam, Netherlands) was used, and the high-frequency line array probe L3–12 color Doppler mode with a probe frequency of 12 MHz was selected, Color Doppler Flow Imaging (CDFI) was simultaneously performed during the examination. Ultrasound examinations were performed by experienced sonographers and orthopedic surgeons together, all ultrasonographic measurements were performed following a standardized protocol, and the same machine was used for all patients.

### 2.3 Method

Color Doppler Ultrasound Examination Method: The direct scanning method is used to apply coupling agent on the skin surface corresponding to the fracture. Then the high-frequency probe is used to take the appropriate position according to the specific condition of the child: the front, medial and lateral (horizontal traction) or the front-back, medial and lateral (hip traction) of the skin surface at the fracture are examined using transverse and longitudinal scanning.

**Figure 1:**
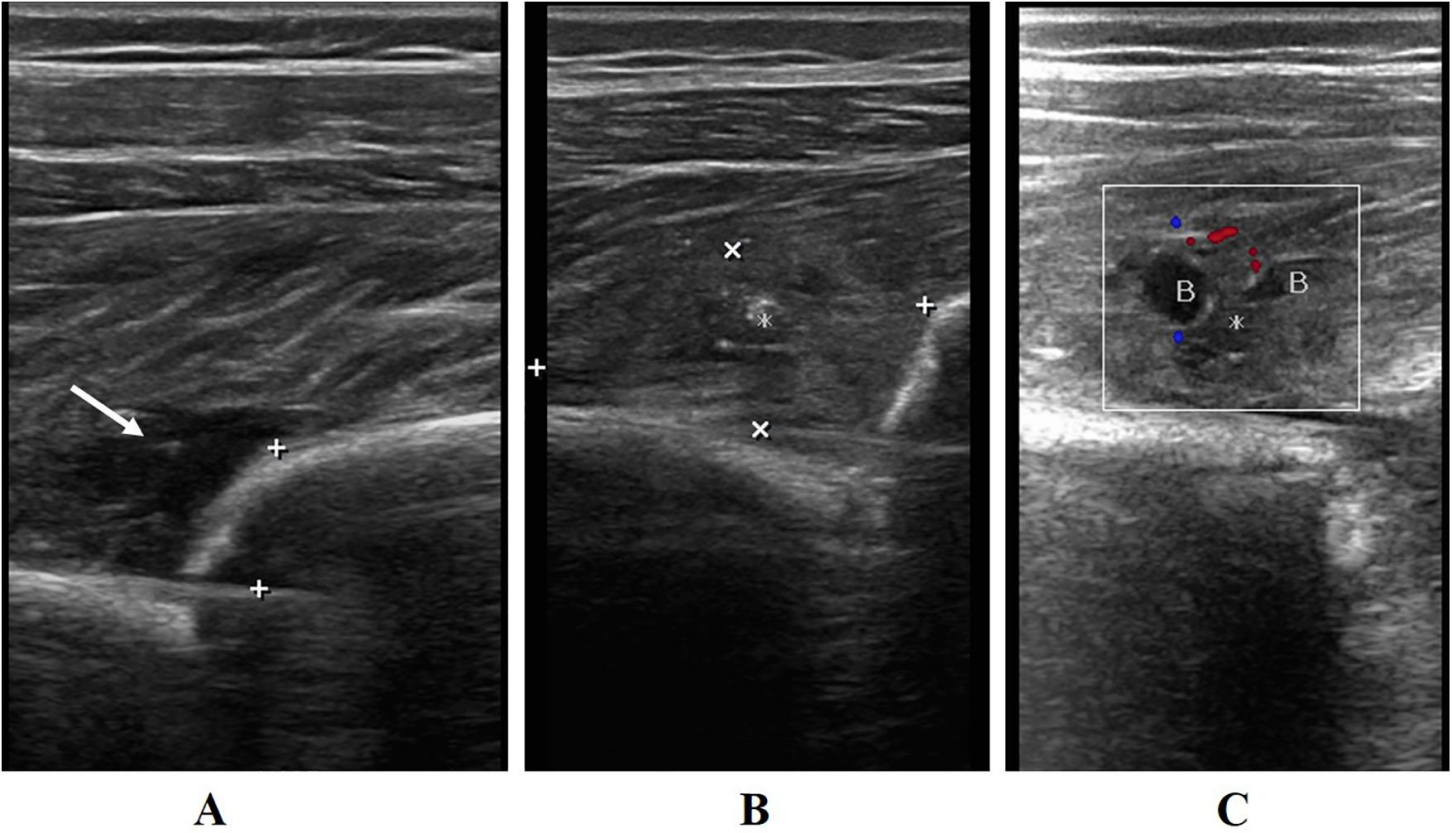
**(A)** longitudinal section of a 4-year-old child with a left middle femur fracture reveals a hematoma (white arrow) at the edge of the fracture (+) on ultrasound. **(B)** Longitudinal section of bone callus length (+) and thickness (×) on ultrasound 1 week after the injury. **(C)** Blood supply **(B)** to the bone callus (*) on Color Doppler ultrasound 1 week after the injury.

Using sonographic images of interrupted continuity of strong echogenic light bands in the bone cortex to clarify the location of the fracture. The edge of the fracture is observed for angulation, displacement, separation and shortening, soft tissue insertion, hematoma formation and muscle fiber dissection around. During each examination, callus thickness at the fracture margin and femoral shaft width were measured on identical sonographic planes; CDFI was selected to monitor the blood supply around the edge of the fracture (Figure 1). The callus-to-femur width ratio (callus thickness/femur width, cm/cm), the callus growth rate (callus thickness/days, cm/d), and vascular Resistance Index (RI) were recorded and calculated. RI is a hemodynamic parameter quantifying downstream vascular resistance, it is calculated as: (PSV-EDV)/PSV, where PSV denotes Peak Systolic Velocity and EDV denotes End-Diastolic Velocity.

At weeks 1, 2, 3, and 4 post-injury, children who met the inclusion criteria underwent examinations at the fracture site using Color Doppler ultrasonography, with subsequent data collection. Ultrasound examiners were blinded to fracture site and week. Fracture location was classified by dividing the anatomical location of the femoral shaft into three equal segments as confirmed by initial radiographs. According to the different sites of femoral shaft fractures, all children were divided into 3 groups for comparison: upper segment, middle segment and lower segment; on the other hand, all children were divided into 4 groups for comparison at 1, 2, 3 and 4 weeks according to different time after injury.

### 2.4 Follow-up

All patients were in traction for 4 weeks and were immobilized in a brace after 4 weeks.

### 2.5 Statistical analysis

SPSS ver.26 (IBM, Armonk, NY) was used for analysis, measurement information denoted by mean ± standard deviation, and One-way ANOVA was used for comparison between multiple groups, the Bonferroni correction was applied for multiple *post-hoc* analyses between weeks, the data of each group passed the chi-square test. The *P* value threshold for significance was set at 0.05.

## 3 Results

A total of 43 cases, 31 males and 12 females, met the inclusion criteria in this prospective observational study. High-frequency ultrasonography was performed in 43 children with femoral shaft fractures, of which 34 children had horizontal skin traction and 9 children had hip skin traction. All children in this study had a satisfactory recovery. Among the 43 children with fractures, there were 13 children with upper femoral shaft fractures, including 8 males and 5 females, the average age is 2.26 ± 1.05 years; 23 children with middle femoral shaft fractures, including 18 males and 5 females, the average age is 2.85 ± 2.01 years; and 7 children with lower femoral shaft fractures, including 5 males and 2 females, the average age is 2.45 ± 0.09 years (Table 1). There was no significant difference in average age between the groups (*p* > 0.05).

**Table 1:** Comparison of basic clinical information between each group.

Group	Gender [*n* (%)]		Age (y)
	Male	Female	
Upper (*n* = 13)	8 (61.5%)	5 (38.5%)	2.26 ± 1.05
Middle (*n* = 23)	18 (78.3%)	5 (21.7%)	2.85 ± 2.01
Lower (*n* = 7)	5 (71.4%)	2 (28.6%)	2.45 ± 0.09
*χ* ^2^	1.156		
*F*			0.437
*p*	0.561		0.650

**Figure 2:**
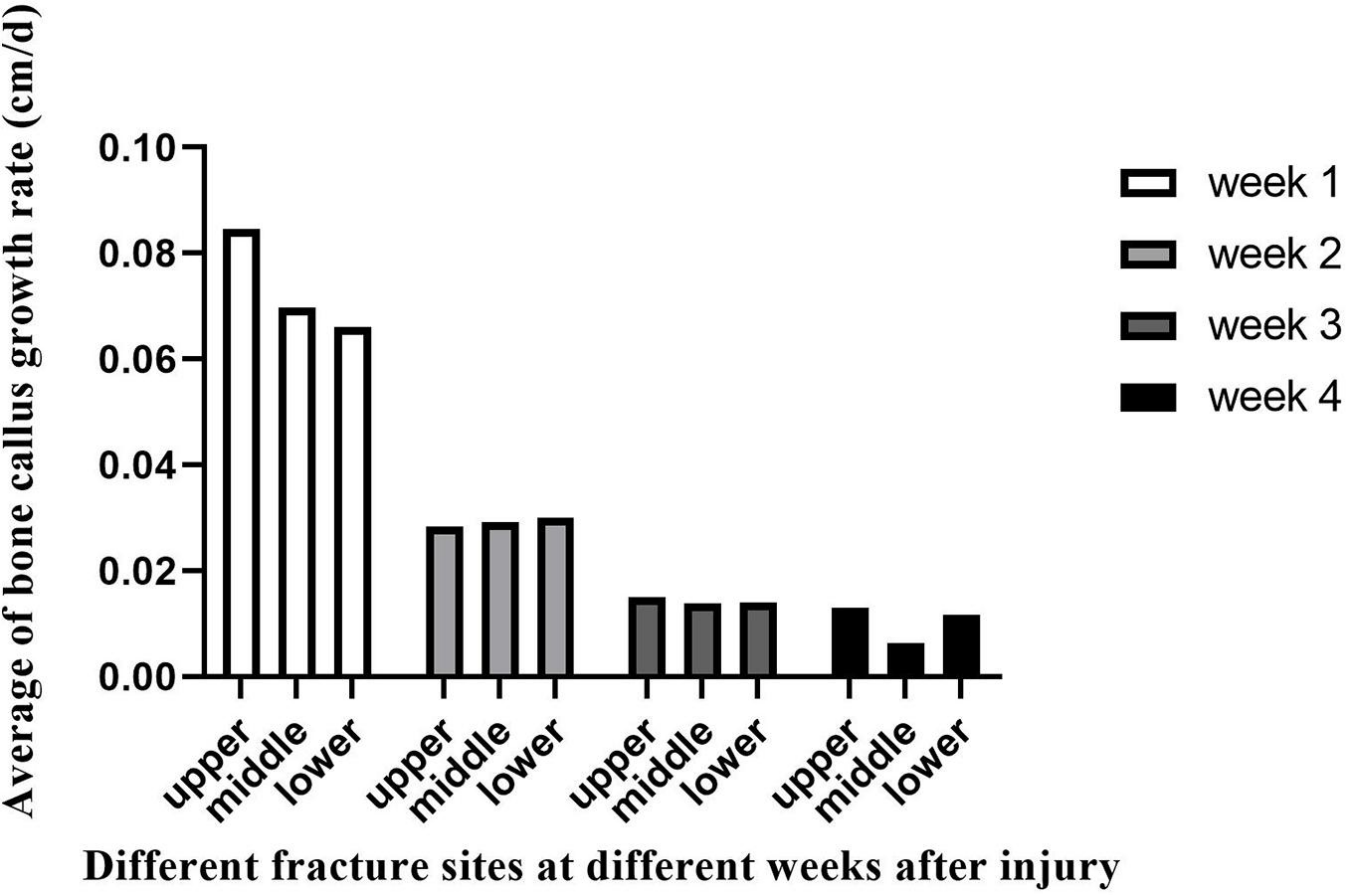
Bone callus growth rate (cm/d) detected at different fracture sites at different weeks after injury.

**Figure 3:**
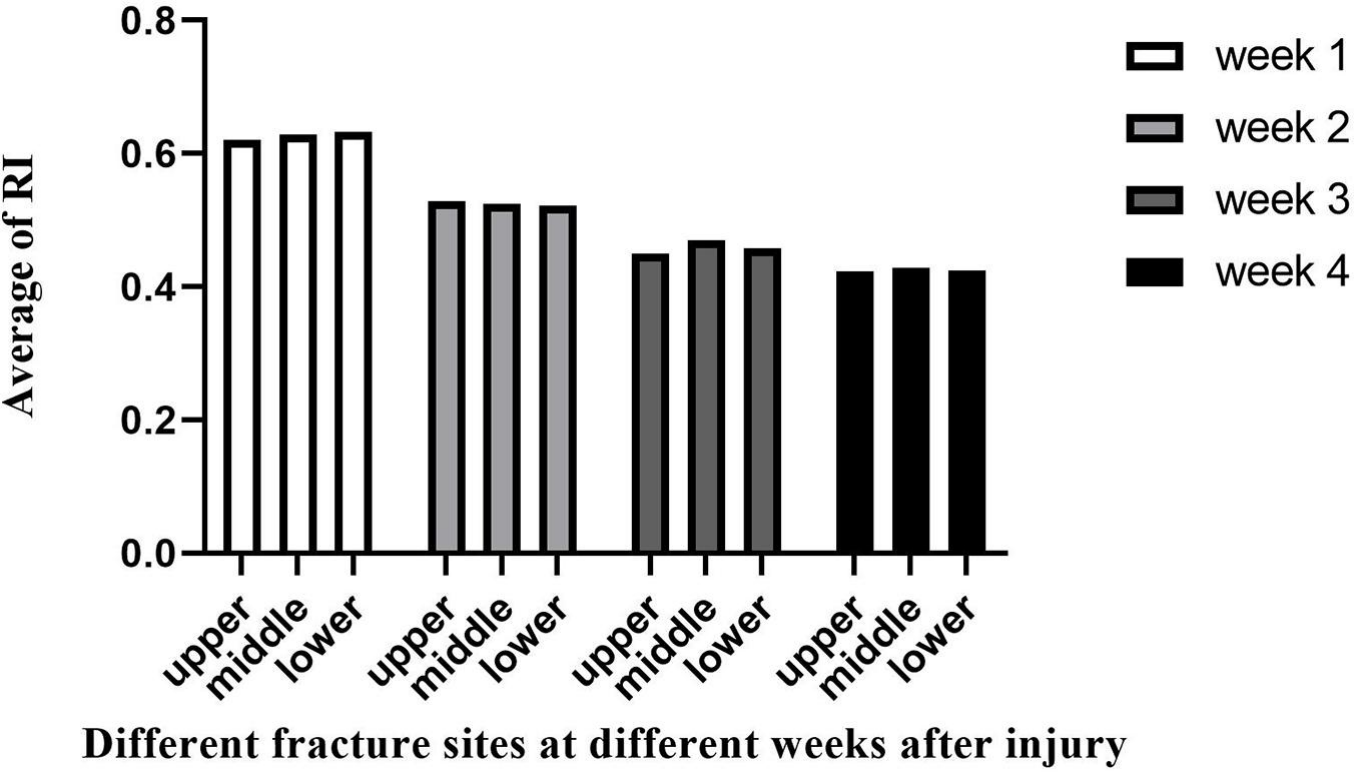
Vascular resistive Index (RI) detected at different fracture sites at different weeks after injury.

As confirmed by Color Doppler ultrasound, there was no statistical difference (*p* > 0.05) in the comparison of the callus-to-femur width ratio (cm/cm), bone callus growth rate (cm/d), and RI in the three groups with femoral fracture sites located in the upper, middle, and lower segments at the same time after injury (Tables 2–5). However, at week 4, we found a difference in callus growth rate (cm/d) between the upper femoral fracture and the middle femoral fracture (Table 5): the callus growth rate (cm/d) of the upper femoral fracture was faster than the middle one. In addition, at the same time after the injury, we found that the trend of callus growth rate (cm/d) and RI between the groups was as follows: the group with relatively faster callus growth rate (cm/d) had a smaller RI, and the group with slower callus growth rate (cm/d) had a larger RI (Figures 2, 3).

**Table 2:** Comparison of examination results between groups at week 1 after injury.

Group	Callus-to-femur width ratio (cm/cm)	Callus growth rate (cm/d)	Vascular resistive index (RI)
Upper	0.40 ± 0.12	0.08 ± 0.03	0.62 ± 0.07
Middle	0.31 ± 0.12	0.07 ± 0.03	0.63 ± 0.12
Lower	0.31 ± 0.09	0.07 ± 0.02	0.63 ± 0.08
*F*	1.852	0.951	0.024
*p*	0.176	0.399	0.976

**Table 3:** Comparison of examination results between groups at week 2 after injury.

Group	Callus-to-femur width ratio (cm/cm）	Callus growth rate (cm/d)	Vascular resistive index (RI)
Upper	0.53 ± 0.12	0.03 ± 0.02	0.53 ± 0.08
Middle	0.45 ± 0.16	0.03 ± 0.03	0.53 ± 0.10
Lower	0.47 ± 0.10	0.03 ± 0.02	0.52 ± 0.07
*F*	1.800	0.007	0.010
*p*	0.323	0.993	0.990

**Table 4:** Comparison of examination results between groups at week 3 after injury.

Group	Callus-to-femur width ratio (cm/cm)	Callus growth rate (cm/d)	Vascular resistive index (RI)
Upper	0.60 ± 0.15	0.01 ± 0.01	0.45 ± 0.10
Middle	0.52 ± 0.18	0.01 ± 0.01	0.47 ± 0.09
Lower	0.55 ± 0.14	0.01 ± 0.02	0.46 ± 0.04
*F*	0.931	0.017	0.185
*p*	0.406	0.983	0.832

**Table 5:** Comparison of examination results between groups at week 4 after injury.

Group	Callus-to-femur width ratio (cm/cm)	Callus growth rate (cm/d)	Vascular resistive index (RI)
Upper	0.67 ± 0.15	0.01 ± 0.01^a^	0.42 ± 0.09
Middle	0.55 ± 0.20	0.01 ± 0.01^b^	0.43 ± 0.08
Lower	0.60 ± 0.15	0.01 ± 0.01	0.43 ± 0.02
*F*	1.463	2.935	0.017
*p*	0.249	0.070	0.983

a Compared with the middle section, ^a^*p* < 0.05.

b Compared with the upper section, ^b^*p* < 0.05.

On the other hand, when compared at 1, 2, 3 and 4 weeks according to different time after injury, statistically significant differences (*p* < 0.05) were detected in the comparison of the callus-to-femur width ratio (cm/cm), callus growth rate (cm/d), and RI in the different time groups (Table 6). When multiple comparisons were performed, we found no statistically significant differences in the callus-to-femur width ratio (cm/cm) between weeks 2 and 3 compared, weeks 3 and 4 compared (*p* > 0.05); no statistically significant differences in the callus growth rate (cm/d) between weeks 3 and 4 (*p* > 0.05); and no statistically significant differences in RI between weeks 3 and 4 (*p* > 0.05).

**Table 6:** Comparison of test results at different weeks after injury.

Group	Callus-to-femur width ratio (cm/cm)	Callus growth rate (cm/d)	Vascular resistive index (RI)
Week 1	0.34 ± 0.12	0.07 ± 0.03	0.64 ± 0.09
Week 2	0.48 ± 0.14^a^	0.03 ± 0.02	0.53 ± 0.09
Week 3	0.55 ± 0.17^b^	0.01 ± 0.01^d^	0.46 ± 0.08^f^
Week 4	0.59 ± 0.18^c^	0.01 ± 0.01^e^	0.43 ± 0.08^g^
*F*	15.187	59.789	36.806
*p*	<0.001	<0.001	<0.001

a Compared with week 3, ^a^*p* > 0.05.

b Compared with week 2, week 4, ^b^*p* > 0.05.

c Compared with week 3, ^c^*p* > 0.05.

d Compared with week 4, ^d^*p* > 0.05.

e Compared with week 3, ^e^*p* > 0.05.

f Compared with week 4, ^f^*p* > 0.05.

g Compared with week 3, ^g^*p* > 0.05.

## 4 Discussion

Fractures are one of the most common injuries in children, multiple repeated examinations are usually required in the treatment of fractures. x-ray examination is widely used for detecting fractures in children. However, the use of x-ray examination has the following disadvantages: (1) It has radiological radiation. (2) Portable x-ray equipment is cumbersome to operate. (3) It cannot be observed dynamically in real time. (4) It is difficult to detect fibrous soft bone callus in early stages (29).

Ultrasonography is a new alternative method. Ultrasound imaging of bone is based on the difference in acoustic impedance between soft tissue and bone, and ultrasound can create a distinct acoustic interface between the two (30, 31). In addition, when scanning soft tissues, bone callus, the edge of the fracture hematomas and other tissues, ultrasound can show more details and have better performance (13, 14, 32). Ultrasonography allows multiple measurements in different axes and angles. It also can clarify the blood supply to the tested area. Compared with adults, children have a thinner subcutaneous fat layer, which is an advantage in using ultrasound. Multiple studies demonstrate the optimal efficacy of ultrasonography for evaluating superficial skeletal structures such as the radius and ulna (33–36). The periosteum of pediatric bones has a stronger osteogenic capacity, so pediatric fractures exhibit accelerated healing compared to adult fractures and with more prominent callus formation (37, 38). During the hematoma mechanization period in the early fracture healing process, the callus produced in the early stage are fibrous callus, which are soft and cannot be visualized by x-ray examination. As time increases, the calcium salt content of the callus gradually increases and the density of the callus gradually increases before they can be visualized by x-ray (29). These studies have confirmed that the appearance of bone callus detected by ultrasonography is significantly earlier than the appearance of bone callus detected by x-ray.

As for the treatment of pediatric femoral fractures, there is no clear consensus has been reached regarding optimal treatment (39–41). For pediatric femoral shaft fractures in older children (generally >5 years of age), surgical management with intramedullary fixation demonstrates effective treatment outcomes, with both elastic stable intramedullary nailing (ESIN) and submuscular plating (SMP) proving to be viable options depending on fracture characteristics and surgical considerations (42, 43). Conservative treatment is an appropriate option for younger children with femoral shaft fractures. Studies have indicated that conservative traction management is the preferred approach for most children under 6 years old with isolated femoral shaft fractures, and can achieve clinically effective outcomes (41, 44–46). This finding is consistent with the age distribution of patients with conservative traction management in our study. In General, the parents of these patients typically involve the following considerations. First, most parents express significant concern that anesthesia or sedation may adversely affect neurocognitive development in younger children. A study pointed out that cognitive deficits can be caused by early postnatal exposure to isoflurane (47). Second, as an invasive treatment method, surgical intervention may result in more trauma and carries potential risks of complications such as surgical site infections, deep bone infections, delayed union or nonunion of fractures, along with the necessity for subsequent procedures to remove internal fixation devices. Third, conservative treatments are generally associated with significantly lower costs compared to surgical intervention (48). On the other hand, the older children with femoral shaft fractures require increased traction force due to higher body weight, which predisposes them to complications such as skin necrosis and joint stiffness. Furthermore, prolonged bed rest during traction therapy inevitably disrupts academic progression and may precipitate psychological disorders in severe cases (49). Consequently, these patients’ parents typically choose other treatment options that mitigate these risks. The younger children with femoral shaft fractures have a strong ability to remodel, conservative traction management can maintain the alignment of the fracture and achieve satisfactory appearance and functional recovery. Multiple examinations are usually required during conservative treatment to avoid overlapping, separation or rotational deformity of the edge of fracture. The use of ultrasound can avoid a lot of radiation which is generated by the use of conventional x-ray examination. Furthermore, ultrasound plays a critical role in the surgical treatment of pediatric fractures, effectively reducing radiation exposure for both patients and the operating team during procedures (50–53).

Ultrasonography can also play a key role in monitoring the bone healing. Ultrasound has a great soft tissue resolution and can clarify the vascularization at an early stage, providing an anticipatory assessment of callus production (54–56). With the continuous development of ultrasound technology, more and more ultrasound detection tools have been applied (57–59). In recent years, a study has also proposed that satisfactory results can be obtained by using ultrasound pitch catch measurements instead of conventional radiography for fracture detection (60). Furthermore, the ongoing refinement of three-dimensional ultrasonography (3D US) opens new avenues for application in fracture assessment (61–63).

**Figure 4:**
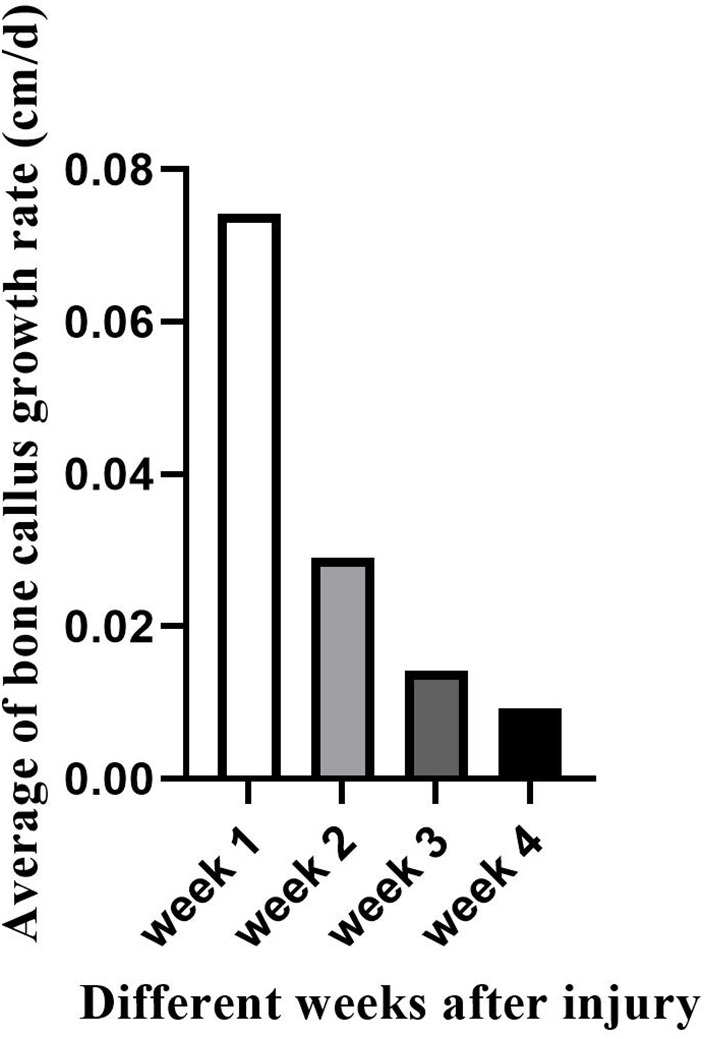
Changes in the bone callus growth rate (cm/d) at different weeks after injury.

**Figure 5:**
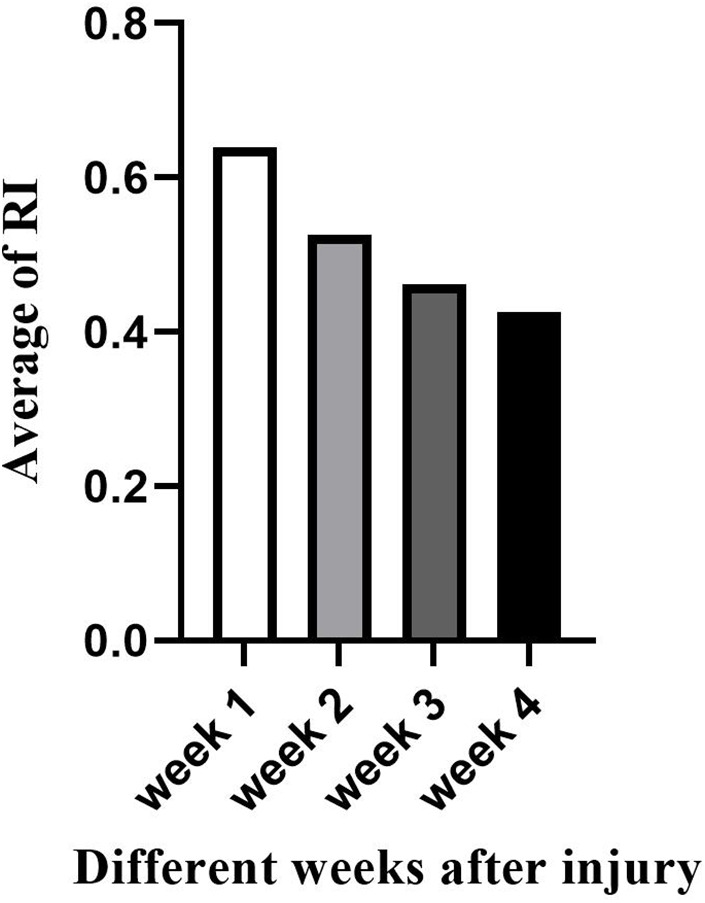
Changes in the vascular resistive Index (RI) at different weeks after injury.

**Figure 6:**
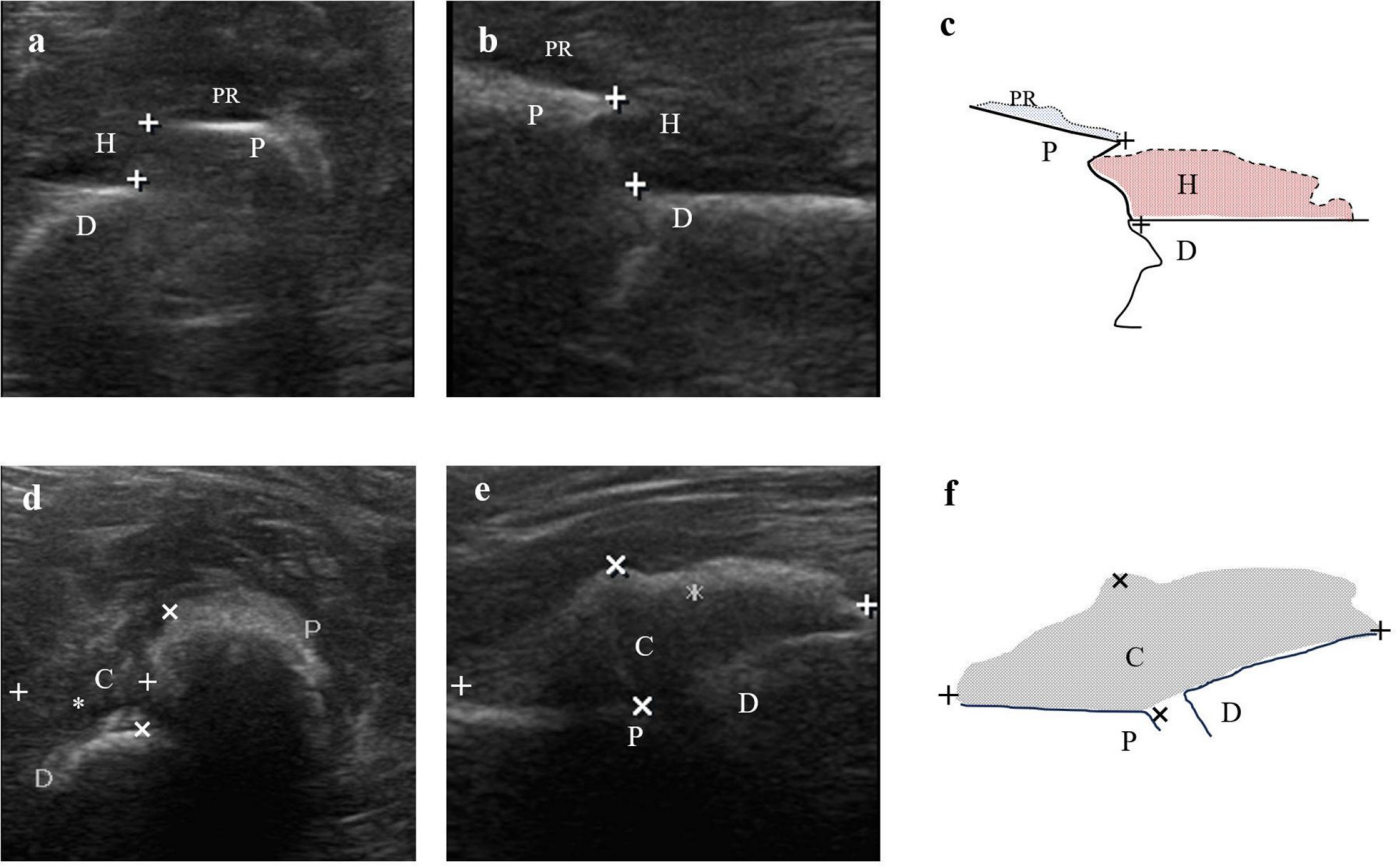
Left middle femur fracture of a 1-year-old boy within 1 day after injury **(a–c)** and after 1 week with conservative treatment **(d–f)**. **(a)** Cross-sectional ultrasound sonogram **(b)** Longitudinal ultrasound sonogram **(c)** Demonstrate the schematic diagram of fracture within 1 day after injury in the longitudinal section **(d)** Cross-sectional ultrasound sonogram **(e)** Longitudinal ultrasound sonogram **(f)** Demonstrate the schematic diagram of fracture after 1 week with conservative treatment in the longitudinal section. C, callus; D, distal end; P, proximal end; H, hematoma; PR, periosteal reaction. The distance between fracture ends [“+” in **(a–c)**]. Callus length [“+” in **(d–f)**]. Callus thickness (×).

In this study, Color Doppler Ultrasound could be better applied to monitor the bone healing process in conservative treatment for pediatric femoral shaft fractures, and we achieved satisfactory results. There was no statistical difference in the comparison of the callus-to-femur width ratio (cm/cm), bone callus growth rate (cm/d), and vascular Resistive Index (RI) in the three groups with femoral fracture sites located in the upper, middle, and lower segments at the same time after injury. This indicated that the recovery of femoral fractures in children in different locations at the same time after injury was essentially the same during the early 4-week healing period under conservative treatment, which could indirectly indicate that there was no significant difference in the degree of femoral vascularization. In comparison, we found that the group with relatively faster callus growth rate (cm/d) had a smaller RI, and the group with slower callus growth rate (cm/d) had a larger RI. This suggested the possibility of a correlation between callus growth rate (cm/d) and RI. On the other hand, when compared at 1, 2, 3 and 4 weeks according to different time after injury, statistically significant differences were detected in the comparison of the callus-to-femur width ratio (cm/cm), callus growth rate (cm/d), and RI in the different time groups. As the fracture healing time gradually increased, both the rate (cm/d) of bone callus growth and the RI gradually decreased (Figures 4, 5). This suggested that ultrasound monitoring allowed for a short-term assessment of the bone healing of femoral shaft fractures in children. When multiple comparisons were performed, we found no statistically significant differences both in the callus-to-femur width ratio (cm/cm), the callus growth rate (cm/d) and RI between weeks 3 and 4. We believed that the main reason was that the callus formation was in the hematoma mechanization period at the early stage of bone healing, the blood supply was abundant, the fibrous callus formation was faster and differed significantly, and gradually decreased as the bone healing time increased (Figure 6).

We acknowledge several limitations in our present study. First, the relatively small sample size (*n* = 43) from a single institution may limit the statistical power and generalizability of our findings. Future multi-center studies with larger cohorts are necessary to validate our results. Second, as an observational study, the allocation to fracture location groups was based on anatomical nature rather than randomization, which could introduce selection bias. Third, the accuracy of ultrasonographic measurements is operator-dependent. Despite using standardized protocols and having examinations performed by experienced sonographers, inter-observer variability was not specifically assessed in this study. Lastly, our follow-up was limited to 4 weeks to assess the early healing phase; longer-term studies are needed to correlate our sonographic findings with functional outcomes and full radiographic union.

Building on our findings and clinical implications, we attempt to propose a structured protocol for integrating ultrasonography into the follow-up of conservative traction managed pediatric femoral shaft fractures, aiming to minimize radiation exposure: (1) Diagnosis and Baseline Assessment: Confirm fracture type and location with radiographs at presentation. (2) Early High-Frequency Ultrasound Monitoring (Weeks 1–4): Perform weekly ultrasound examinations focusing on bone callus growth rate (cm/d) and vascular Resistive Index (RI) at the fracture site. During this phase, ultrasound can effectively replace routine weekly radiographs. (3) Mid-term Evaluation (Weeks 4–6): If ultrasound demonstrates robust callus bridging and a steadily decreasing RI, radiographs can be safely omitted. Obtain a radiograph only if there is clinical or sonographic concern for delayed healing. (4) Final Healing Assessment: When ultrasound indicates mature callus formation and clinical examination is stable, a final confirmatory radiograph can be obtained to document radiographic union. This protocol has the potential to reduce cumulative radiation exposure during follow-up, which is particularly beneficial for the radiation-sensitive pediatric population.

However, the use of ultrasound to examine pediatric fractures also has limitations such as poor ultrasound image in localized locations, limitations by the examination site, and possible artifacts (64). The utilization of ultrasonography for fracture detection necessitates enhanced training protocols and ongoing technical optimization to ensure diagnostic accuracy (65). In addition, the operation process is more time consuming for both the operators and the patients, which makes the examination more difficult. Taken together, current evidence suggests that ultrasound presents a clinically useful modality for the evaluation of common long bone fractures in children, particularly in settings like emergency departments and primary care, though further standardization of diagnostic criteria is needed. A recent prospective cohort study demonstrates that early diagnosis and surgical intervention (within 48 h) for pediatric femoral fractures significantly reduces operative time, improves clinical outcomes, and decreases the incidence of major complications including avascular necrosis and growth disturbance (66). Several studies also have shown that ultrasound can be better applied in emergency medicine (67–71). In emergency rescue, disaster relief and other unexpected situations, or for children with fractures who are not easily transported, the use of ultrasound for fracture examination is acceptable. Several scholars have systematically summarized the clinical context, indications, and benefits of fracture ultrasound and provided recommendations for its rational application (31). Standardizing imaging protocols, establishing validated reference standards, and conducting large-scale randomized trials will be crucial for the comprehensive integration of ultrasonography into future clinical practice guidelines for fracture management.

The great soft tissue resolution of ultrasound distinguishes it from conventional examinations for fractures. Its exceptional compatibility with pediatric fracture will attract more attention from scholars. It is believed that in the near future, the application of ultrasound in pediatric fractures will see further development.

## Data Availability

The original contributions presented in the study are included in the article/Supplementary Material, further inquiries can be directed to the corresponding author.
